# Supplementary material for: Efficient Cytotoxicity of Recombinant Azurin in Escherichia coli Nissle 1917-Derived Minicells against Colon Cancer Cells
Source: Bioengineering (Basel). 2023 Oct 13;10(10):1188. doi: 10.3390/bioengineering10101188 (PMC10603951; doi:10.3390/bioengineering10101188)
Supplement: Supplementary file 1 [file bioengineering-10-01188-s001.zip › bioengineering-2445693-supplementary.pdf]

Supplementary information

I. Primers

MinCD-R1:

TTTCTTCCGCGAGAGAAAGAAATCGAGTAATGCCATAACTCTCCTTCTTAAAGTTAAACAAATTAGAAAAACTCATCGAGCATC

MinCD-F1:

AGGATGTCAAACACGCCAATCGAGCTTAAAGGCAGTAGCTTCACTTTATATGAGCCATATTCAACGGGAA

CT26-F(GAPDH):

GGTCCCAGCTTAGGTTCAT

CT26-R(GAPDH):

CCCAATACGGCCAAATCCGT

HT29-F(GAPDH):

GGTGACTAACCCTGCGCTC

HT29-R(GAPDH):

CCCAATACGACCAAATCAGAGAAT

CT26-F(p53):

TCCGAAGACTGGATGACTGC

CT26-R(p53):

GATCGTCCATGCAGTGAGGT

HT29-F(p53):

CTTCCCTGGATTGGCAGC

HT29-R(p53) :

TTTCAGGAAGTAGTTTCCATAGGT

II. Suquences

MinE(blue color), RBS(red color), KANA(green color), MinC(black color)

TTATTTCAGCTCTTCTGCTTCCGGTAAGGTCACGTTCAGCTCAAGAATAGAAATATCGCCATCTTTTTGCTCAAGCTGTACGGTTACCATCTCAGGATCAATTTGTACATATTTACAAATGACCTCAAGAATATCTTTACGCAACTGCGGCAGATAATGCGGTTCTGCATCGCTGCGACGGCGTTCAGCAACAATAATCTGCAACCGTTCTTTTGCAATGTTGGCTGTGTTTTTCTTCCGCGAGAGAAAGAAATCGAGTAATGCCATAACtctccttcttaaagttaaacaaattagaaaaactcatcgagcatcaaatgaaactgcaatttattcatatcaggattatcaataccatatttttgaaaaagccgtttctgtaatgaaggagaaaactcaccgaggcagttccataggatggcaagatcctggtatcggtctgcgattccgactcgtccaacatcaatacaacctattaatttcccctcgtcaaaaataaggttatcaagtgagaaatcaccatgagtgacgactgaatccggtgagaatggcaaaagtttatgcatttctttccagacttgttcaacaggccagccattacgctcgtcatcaaaatcactcgcatcaaccaaaccgttattcattcgtgattgcgcctgagcgagacgaaatacgcgatcgctgttaaaaggacaattacaaacaggaatcgaatgcaaccggcgcaggaacactgccagcgcatcaacaatattttcacctgaatcaggatattcttctaatacctggaatgctgttttcccggggatcgcagtggtgagtaaccatgcatcatcaggagtacggataaaatgcttgatggtcggaagaggcataaattccgtcagccagtttagtctgaccatctcatctgtaacatcattggcaacgctacctttgccatgtttcagaaacaactctggcgcatcgggcttcccatacaatcgatagattgtcgcacctgattgcccgacattatcgcgagcccatttatacccatataaatcagcatccatgttggaatttaatcgcggcctagagcaagacgtttcccgttgaatatggctcatATAAAGTGAAGCTACTGCCTTTAAGCTCGATTGGCGTGTTTGACAT
